# Molecular Cloning and Copy Number Variation of a Ferritin Subunit (Fth1) and Its Association with Growth in Freshwater Pearl Mussel *Hyriopsis cumingii*

**DOI:** 10.1371/journal.pone.0022886

**Published:** 2011-07-27

**Authors:** Zhiyi Bai, Yiming Yuan, Genhua Yue, Jiale Li

**Affiliations:** 1 Key Laboratory of Exploration and Utilization of Aquatic Genetic Resources, Shanghai Ocean University, Ministry of Education, Shanghai Ocean University, Shanghai, China; 2 E-Institute of Shanghai Universities, Shanghai Ocean University, Shanghai, China; 3 Molecular Population Genetics Group, Temasek Life Sciences Laboratory, National University of Singapore, Singapore, Singapore; University of Minnesota, United States of America

## Abstract

Iron is one of the most important minor elements in the shells of bivalves. This study was designed to investigate the involvement of ferritin, the principal protein for iron storage, in shell growth. A novel ferritin subunit (Fth1) cDNA from the freshwater pearl mussel (*Hyriopsis cumingii*) was isolated and characterized. The complete cDNA contained 822 bp, with an open reading frame (ORF) of 525 bp, a 153 bp 5′ untranslated region (UTR) and a 144 bp 3′ UTR. The complete genomic DNA was 4125 bp, containing four exons and three introns. The ORF encoded a protein of 174 amino acids without a signal sequence. The deduced ferritin contained a highly conserved motif for the ferroxidase center comprising seven residues of a typical vertebrate heavy-chain ferritin. It contained one conserved iron associated residue (Try27) and iron-binding region signature 1 residues. The mRNA contained a 27 bp iron-responsive element with a typical stem-loop structure in the 5′-UTR position. Copy number variants (CNVs) of Fth1 in two populations (PY and JH) were detected using quantitative real-time PCR. Associations between CNVs and growth were also analyzed. The results showed that the copy number of the ferritin gene of in the diploid genome ranged from two to 12 in PY, and from two to six in JH. The copy number variation in PY was higher than that in JH. In terms of shell length, mussels with four copies of the ferritin gene grew faster than those with three copies (*P*<0.05), suggesting that CNVs in the ferritin gene are associated with growth in shell length and might be a useful molecular marker in selective breeding of *H. cumingii*.

## Introduction

As a cofactor in many biochemical reactions, the trace element iron is important to all living organisms through its role in regulating metabolism, electron transport, oxidative phosphorylation and DNA biosynthesis [1]. However, excess labile iron is detrimental due to its participation in oxidation-reduction reactions that generate harmful free radicals. Therefore, iron homeostasis is important in living cells [1], [2]. Ferritin, a major iron storage protein in living cells, plays a crucial role in intracellular iron homeostasis. Ferritin has 24 subunits, which form a hollow shell and can store up to 4,500 iron (III) atoms [3]. There are two types of ferritin subunit: the heavy chain (H) and the light chain (L). H-ferritin subunits occur in multiple forms in animals, plants and bacteria, while L-ferritin subunits are usually restricted to vertebrates [4], [5]. Ferritin has been shown to be involved in many physiological activities such as development [6], [7], [8], immunity [9], [10] and other cellular mechanisms [11], [12], [13].

Among bivalves, ferritin genes from the pacific oyster (*Crassostrea gigas*), pearl oyster (*Pinctada fucata*), bay scallop (*Argopecten irradians*) and clam (*Meretrix meretrix*) have been characterized [14], [15], [16], [17]. In *M. meretrix*, ferritin is reported to play a role in larval shell development [16]. In *P. fucata*, studies suggest that ferritin is involved in adult shell formation [17]. However, understanding of ferritin gene sequence variation and the biological function of ferritins is limited to freshwater bivalve species. *Hyriopsis cumingii* is a freshwater bivalve that is widely distributed in China and is commercially the most important mussel species exploited for freshwater pearl production in this country [18]. In this study, the full length cDNA and genomic DNA of one ferritin H subunit (Fth1) of *H. cumingii* were isolated and characterized.

Genetic variation takes many forms, ranging from large, microscopically visible chromosome anomalies to single nucleotide changes. Copy number variants (CNVs) represent segments of DNA larger than 1 kb present at in variable copy numbers in comparison with a reference genome [19]. They can be responsible for altered gene expression [20] leading to striking phenotypic variance, including disease associated traits [21], [22]. CNVs have been reported to be present genome-wide not only in humans [23], but also in chimpanzees [24], mice [25], nematodes [26], fruit flies [27] and pigs [28]. However, no study of the extent and impact of CNVs in the molluscan genome has been made until now. We investigated whether the copy number of the ferritin gene differed between a wild population and a cultured population, and whether a functional relationship existed between copy number and growth in *H. cumingii*. To our knowledge, this is the first study of gene copy number variation in freshwater bivalves.

## Results

### cDNA and genomic organization of ferritin gene in *H. cumingii*

**Figure 1:**
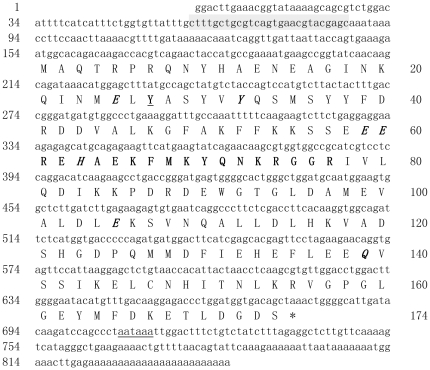
Complete cDNA sequence and deduced amino acid sequence of *H. cumingii* ferritin. The IRE in the 5′ UTR is shaded. Seven residues in bold and italics represent a putative active site of ferroxidase. The iron associated residue Tyr27 is in bold and underlined. Iron-binding region signature 1 is in bold. The star represents the stop codon. The poly(A) signal is underlined.

An 822 bp cDNA of a ferritin subunit (Fth1), containing a 525 bp open reading frame (ORF), a 153 bp 5′ UTR and a 144 bp 3′ UTR, was isolated from *H. cumingii* (Fig. 1). A putative polyadenylation signal (ATTAAA) was recognized at the nucleotide position 708–713 (Fig. 1). The translated amino acid sequence comprised 174 residues with a calculated molecular mass of 20.19 kDa and an isoelectric point of 5.08. SignalP software analysis found no signal peptide. The genomic structure of the ferritin gene was determined by sequencing the genomic fragment amplified by PCR. From the first transcription initiation site, the *H. cumingii* g-ferritin gene extended 4125 bp to the end of the 3′ UTR and contained four exons and three introns. All of the 5′ and 3′ ends of the introns showed canonical splicing motifs (GT/intron/AG).

### An iron-responsive element (IRE) and a stem-loop structure (SLS) in the ferritin subunit of *H. cumingii*

**Figure 2:**
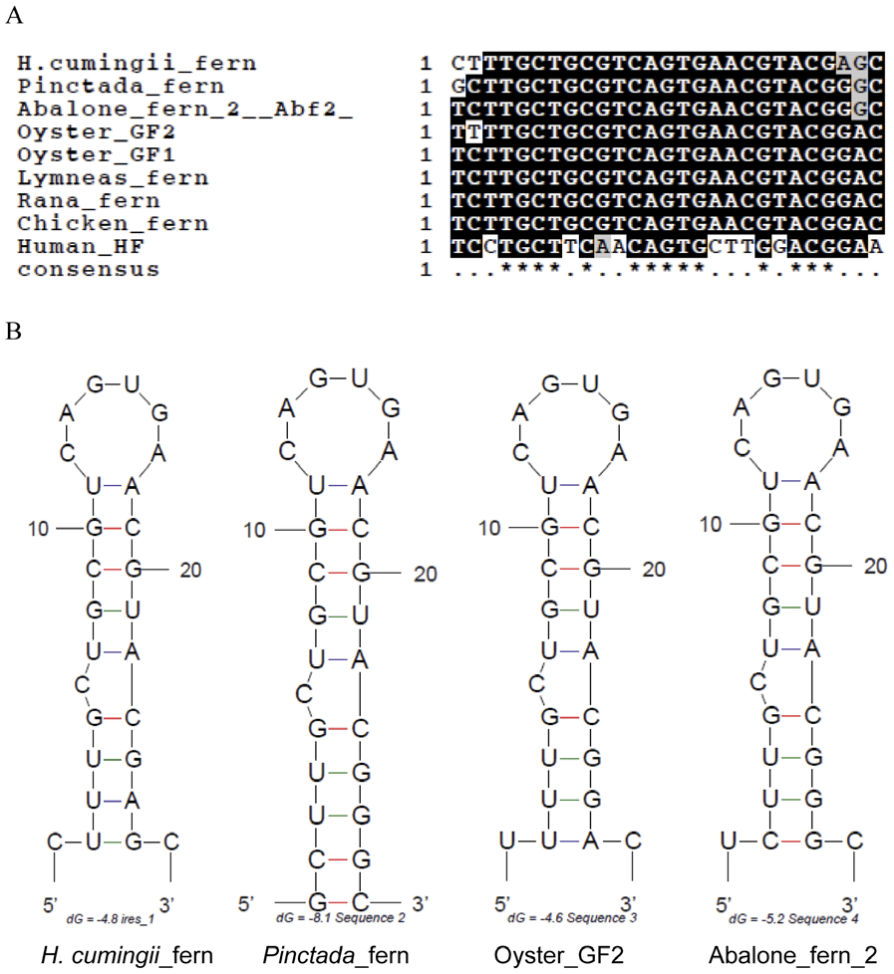
Alignments of IRE and SLS of *H. cumingii* with those of other species. (A) Alignment of the IRE with selected ferritin IREs. Consensus residues are shaded and identical residues are in bold. Freshwater pearl mussel (*H. cumingii*), Fth1; pearl oyster (*P. fucata*), AAQ12076.1; abalone (*Haliotis discus hannai*), DQ821494; pacific oyster (*C. gigas*), AY321299 and AY321300; bullfrog (*Rana catesbeiana*), M12120; great pond snail (*Lymnaea stagnalis*), X56778; chicken (*Gallus gallus*), NM205086; and human (*Homo sapiens*), NM002032. (B) A comparison of the IRE SLS of *H. cumingii* and known ferritins of *P. fucata* (AAQ12076.1), *C. gigas* (AY321300) and *H. discus* (DQ821494). Typical CAGUGA loop structure residues and bulged cysteines are seen in all four species.

An SLS (5′-ctttgctgcgtcagtgaacgtacgagc-3′) in the 5′ UTR of ferritin subunit cDNA of *H. cumingii* was recognized as an iron-responsive element (or iron regulatory element, IRE) (Fig. 1). IRE alignment showed a high identity between *H. cumingii*, other species of mollusk and chicken, with only three or four bases different in their flank sequences (Fig. 2A). The most conserved motif in the IRE, 5′-CAGTGA-3′, is in good agreement between species, with the exception of humans (Fig. 2A). This putative IRE could be folded into a typical secondary SLS (Fig. 2B) matching all characteristics of IREs, including the six nucleotide loop 5′-CAGUGA-3′’, the proximal stem of five paired bases followed by a bulged cysteine, and the six nucleotide bottom stem.

### Homology comparison and phylogenetic analysis of ferritin in mollusks

**Figure 3:**
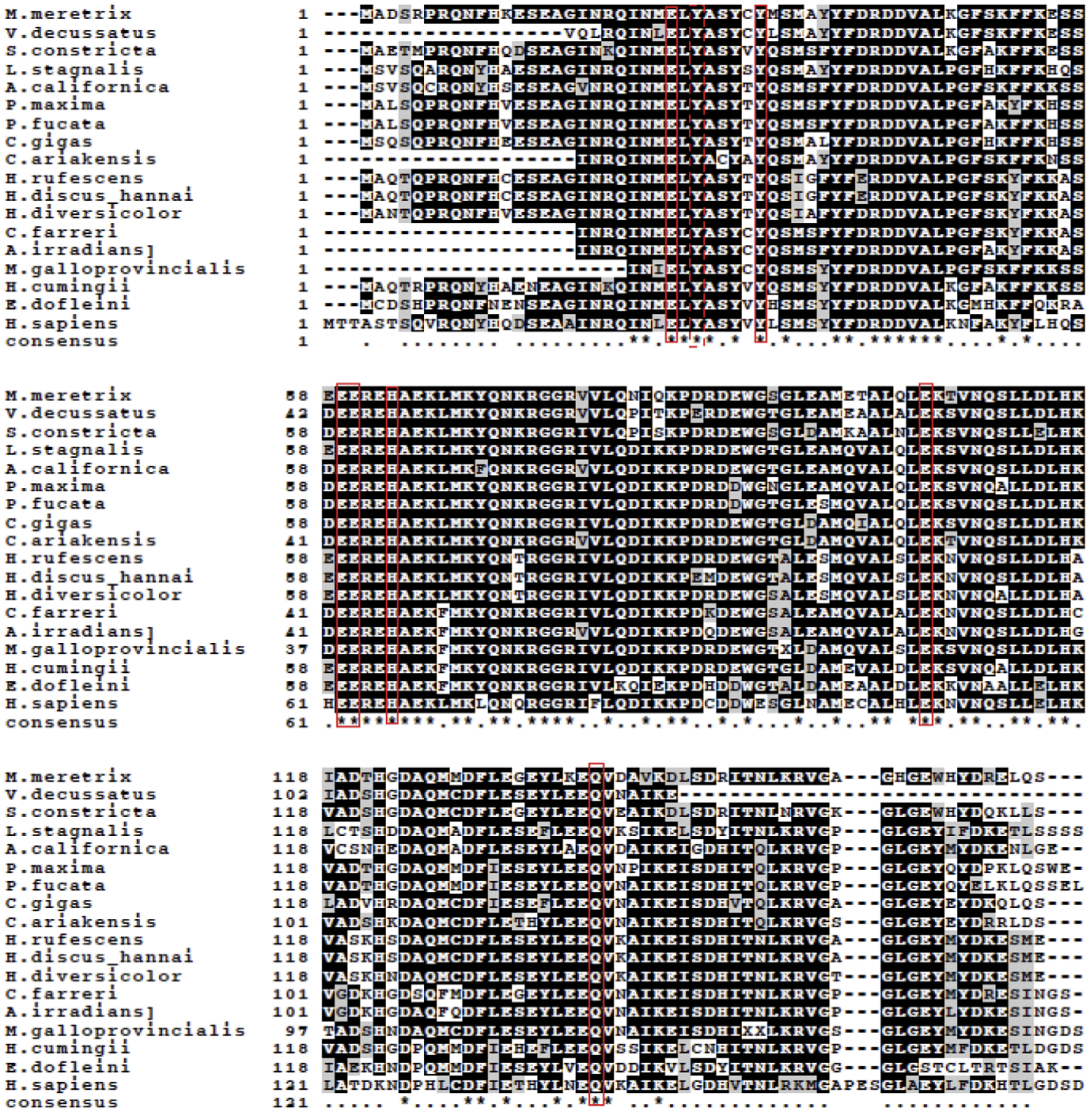
Multiple alignments of *H. cumingii* ferritin with homolog from other mollusk species and human. Identical amino acid residues are darkly shaded, similar amino acids are lightly shaded, unrelated residues have a white background. *C. gigas*, AAP83794.1; *Crassostrea ariakensis*, ABE99842.1; *P. fucata*, AAQ12076.1; *Pinctada maxima*, ACS72281.1; *Chlamys farreri*, AAV66906.1; *A. irradians*, AAV66907.1; *Mytilus galloprovincialis*, ACM86786.1; *Sinonovacula constricta*, ACZ65230.1; *Venerupis decussatus*, ACB38006.1; *M. meretrix*, AAZ20754.1; *Haliotis rufescens*, ACZ73270.1; *H. discus hannai*, ABH10672.1; *H. diversicolor*, ABY87353.1; *Lymnaea stagnalis*, P42577.2; *Aplysia californica*, ABF21074.1; *Enteroctopus dofleini*, AAD29639.1; *Homo sapiens*, AAA52437.1.

**Figure 4:**
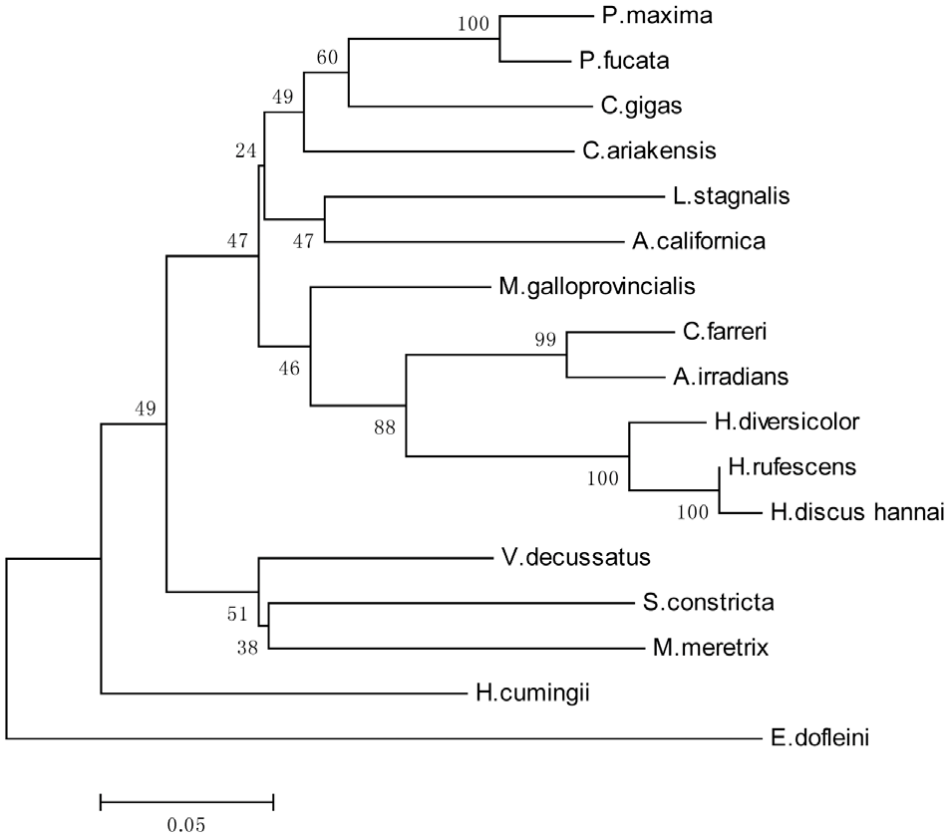
Neighbor-joining phylogenetic tree of ferritins from mollusk species. *C. gigas*, AAP83794.1; *C. ariakensis*, ABE99842.1; *P. fucata*, AAQ12076.1; *P. maxima*, ACS72281.1; *C. farreri*, AAV66906.1; *A. irradians*, AAV66907.1; *M. galloprovincialis*, ACM86786.1; *S. constricta*, ACZ65230.1; *V.s decussatus*, ACB38006.1; *M. meretrix*, AAZ20754.1; *H. rufescens*, ACZ73270.1; *H. discus hannai*, ABH10672.1; *H. diversicolor*, ABY87353.1; *L. stagnalis*, P42577.2; *A. californica*, ABF21074.1; *E. dofleini*, AAD29639.1; *E. dofleini*, AAD29639.1.

Homologous sequence alignment using ClustalW revealed uniformly high similarities between Fth1 of *H. cumingii* and ferritin of other species, ranging from 64% (*H.sapiens*) to 81% (*M.galloprovincialis*) (Table 1). Homologous sequence alignment also indicated seven amino acid residues, identified as metal ligands at the H-specific ferroxidase center in mammalian ferritins, that were completely conserved in the ferritin subunit. They were Glu25, Tyr32, Glu59, Glu60, His63, Glu105 and Gln139. A potential biomineralization residue, Tyr27, was also conserved (Fig. 3). Phylogenetic analysis based on ferritin subunit amino acid sequences showed *H. cumingii* to be distant from seawater bivalves (Fig. 4).

**Table 1:** Pairwise similarities between Fth1 of *H. cumingii* and ferritin genes of other species as specified.

*Ferritin genes of other Species*	*Similarities to Fth1 of H. cumingii*
*C. gigas*, AAP83794.1;	77
*C. ariakensis*, ABE99842.1	77
*P. fucata*, AAQ12076.1	77
*P. maxima*, ACS72281.1	78
*C. farreri*, AAV66906.1	77
*A. irradians*, AAV66907.1	76
*M. galloprovincialis*, ACM86786.1	81
*S. constricta*, ACZ65230.1	77
*V. decussatus*, ACB38006.1	78
*M. meretrix*, AAZ20754.1	74
*H. rufescens*, ACZ73270.1	75
*H. discus hannai*, ABH10672.1	74
*H. diversicolor*, ABY87353.1	75
*L. stagnalis*, P42577.2	80
*A. californica*, ABF21074.1	76
*E. dofleini*, AAD29639.1	70
*H. sapiens*, AAA52437.1	64

### Copy number variation of ferritin in wild and cultured populations

**Figure 5:**
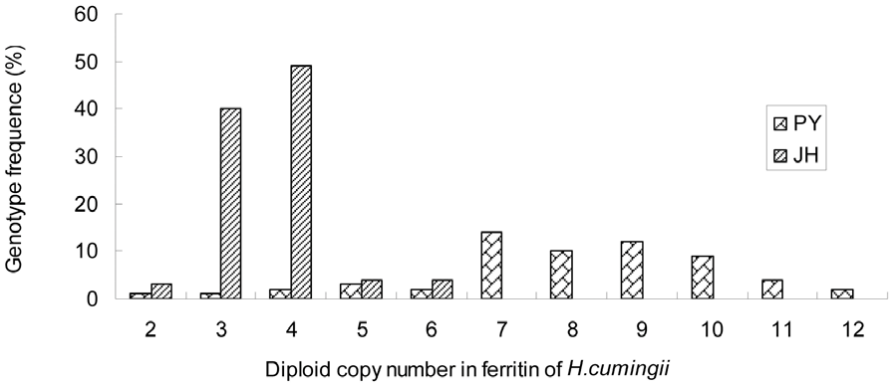
Genotype frequencies of diploid copy number in ferritin gene in wild (PY) and cultured (JH) populations of *H. cumingii*.

Extensive variations in the copy number of the ferritin gene were observed in both wild (PY) and cultured (JH) populations. There were more copy number variations in the wild population than in the cultured population, ranging from two to 12 and two to six respectively. In the wild population, seven, eight, nine, and 10 CNVs were the principal genotypes (representing 23.3%, 16.7%, 20.0% and 15.0% respectively); other CNVs were less common. In the cultured population, three and four CNVs were the principal genotypes (40.0% and 49.0% respectively); again, other CNVs were less common (Fig. 5).

### Analysis of association between CNVs and growth

The growth of individuals from the JH population was recorded before genotyping of the ferritin gene. Differences between individuals with three and those with four CNVs were analyzed. The t-test revealed that, in terms of shell length, mussels with four copies of ferritin grew faster than those with three copies (*P*<0.05), but no significant differences were observed in shell width and body weight (Table 2).

**Table 2:** Growth *H. cumingii* with different ferritin copy numbers (mean ± SE).

Copy Number	Individuals	Shell Length	Shell Width	Weight
3	40	133.37±1.29^a^	33.23±1.35^a^	256.85±15.66^a^
4	49	136.57±1.30^b^	32.98±0.74^a^	267.39±9.97^a^

Same superscript represents no obvious difference; different superscript represents obvious difference (*P*<0.05).

## Discussion

In this study, we cloned one ferritin gene in an important freshwater pearl mussel species, *H. cumingii*. According to the bioinformatics analysis, this ferritin is likely to be a homolog of vertebrate H-ferritin. This is further supported by the finding of a putative ferroxidase site in the deduced peptide, where seven amino acid residues were located in the same positions as in vertebrate H-ferritin [3]. The Tyr27 in ferritin is important for iron binding [29], enabling iron biomineralization. An IRE was identified, which is important for the regulation of ferritin translation [5]. When cellular iron is low, the iron regulatory protein (IRP) can repress the expression of ferritin by binding to the SLS of IRE. When cellular iron is high, IRP unbinds from the IRE and ferritin mRNA translates ferritin to store extra iron [3], [30]. The IRE and IRP have been greatly conserved during evolution [31]. The IRE of *H. cumingii* ferritin cDNA is homologous with those of other species (Fig. 2). In this case, the ferritin might be regulated on a mainly translational level.

Iron is an important trace element in the prismatic and nacreous layers of bivalve shells [32], [33]. The mantle epithelial cells of bivalves have been shown to accumulate iron and other elements in lysosomes, which participate actively in the incorporation of metals into the shell [34], [35]. Calcified concretions containing calcium, iron and other metals have been described in the mantles of *Hyridella depressa* and *Margaritifera margaritifera*, which were proposed to accumulate metals and contribute to shell formation [36], [37], [38]. To further understand the role of ferritin in shell formation by *H. cumingii*, the relationship between genotype and phenotype is an effective investigation. CNVs are present genome-wide and usually associated with phenotypic variance [39]. In humans, the ferritin gene copy number varies widely. In this study, ferritin CNVs and their association with the growth of *H. cumingii* were determined.

Recently, real-time quantitative PCR has become an attractive method for gene copy number measurements, being fast, precise and reproducible and having a high throughput [40], [41], [42], [43]. In contrast, traditional methods for measuring DNA copy numbers, such as fluorescence in situ hybridization, are difficult to perform with a high throughput and fail to detect small deletions/duplications [44], [45]. Southern blotting and chromosomal comparative genomal hybridization require relatively large amounts of genomic DNA, and Southern blotting is time consuming and labor intensive [44], [45]. Multiplex amplification and probe hybridization (MAPH) and multiplex ligation dependent probe amplification are promising new methods to detect dosage changes. However, neither is able to detect translocations in balanced form [45]. Furthermore, MAPH can detect a large relative difference in copy number, such as one copy instead of two, but it is not sufficiently sensitive to discriminate between high copy numbers [46]. In this study, a method of real-time quantitative PCR to detect copy number polymorphisms in *H. cumingii* ferritin was established.

Compared with other real-time quantitative PCR methods reported previously, the method introduced here was improved by a minor modification. To estimate copy number using a comparative cycle threshold (CT) method, it is necessary to identify a single-copy gene as the control. However, only a small number of genomic and cDNA sequences are known for *H. cumingii*, and no single-copy gene has yet been identified. Justen et al. developed an efficient method for amplifying single-copy nuclear loci by designing a primer anchored in the 3′ UTR [47]. In this study, we designed primers in the 3′ UTR to amplify single-copy nuclear loci as control genes, and in the conserved region of exon2 for target genes. This method will provide a powerful tool for screening copy number polymorphisms in non-model species.

Using this method, the CNVs of ferritin in wild and cultured populations were determined. To our knowledge, these are the first genotype data obtained for CNVs in freshwater bivalves. Our finding that there were more copy number variations in the wild population than in the cultured population is in agreement with other results obtained by simple sequence repeats and other markers in our laboratory [18], [48]. In the wild population, seven, eight, nine, and 10 copy number variations were the principal genotypes, which suggested the mussels with higher copy number in ferritin would be easier to survive in lake, and implied that ferritin dosage may convey a growth or resistance advantage. In the cultured population, three and four copy number variations were the principal genotypes. It is most possible that the cultured population constructed on several parent mussels with lower copy number in ferritin. One female mussel can release at least 2–4 hundred thousand of offsprings, so offsprings reproduced by several mussels are sufficient for the production demand of one pearl mussel farm. In this study, preliminary analysis of the association between CNVs and growth suggested that ferritin may be involved shell formation, which may be improved by a greater ferritin copy number. However, this is only the first step towards an understanding of the functions of the ferritin gene in shell formation. Further studies will help to discover the role of the ferritin copy number polymorphisms.

In conclusion, we cloned a ferritin gene (Fth1) in an important freshwater pearl mussel species *H. cumingii*, developed an approach to estimate ferritin gene CNVs and detected a potential association between CNVs and growth, for the first time in a freshwater bivalve. Further studies on the mechanisms underlying the association between growth and ferritin CNVs are required.

## Materials and Methods

### Ethics Statement

Collecting mussels of wild stock from Duchang County of Jiangxi Province was permitted by Department of Fishery of Poyang Lake (DFPL2008WAC11). All handling of mussels was conducted in accordance with the guidelines on the care and use of animals for scientific purposes set up by the Institutional Animal Care and Use Committee (IACUC) of Shanghai Ocean University, Shanghai, China.

### Samples and DNA extraction

Adult *H. cumingii* were randomly collected from wild and cultured stocks in 2008. The wild stock was collected from Duchang County of Jiangxi Province, around Poyang Lake (29.2°N 116.2°E). The cultured stock was Jinhua (JH) cultured stock from the Weiwang pearl farm of Jinhua City, Zhejiang Province. Sixty adults were sampled randomly from the wild stock. From the cultured stock, 100 3-year-old individuals that had been produced at the same time and cultured in the same pond were sampled. Shell length, shell width and body weight were recorded for each individual from the Jinhua stock. Mantle samples from each individual were stored in 95% ethanol until DNA extraction. Genomic DNA was isolated from the tissues using a Genomic DNA Extraction Kit (Innogent, Shenzhen, China) according to the manufacturer's instructions. Compared with other DNA extraction kits, this kit was recommended for the acquisition of a high quality DNA template, which was important for the reliability of this experiment. The concentration of the extracted DNA was estimated spectrophotometrically. The DNA was diluted in 10 mM pH 8.5 Tris buffer to a concentration of approximately 50 ng/µL and stored at −20°C until use.

### Isolation of full length cDNA and genomic DNA

One expressed sequence tag (EST) of ferritin (GW694187) was originally identified from the mantle cDNA library constructed in our laboratory [49]. The full length cDNA of the ferritin was identified by further sequencing of the original EST clone using a primer walking approach. The complete genomic DNA sequence of the ferritin was amplified using PCR with primers (gFER-F1 and gFER-R1, Table 3) by long distance PCR, and sequenced using Bigdye chemicals (Applied Biosystems). Exons and introns were identified by aligning the cDNA and genomic DNA sequences of ferritin using Sequencher (Gene Codes). The full length cDNA sequence and genomic DNA of ferritin were submitted to GenBank with accession numbers HQ896721 and HQ896722, respectively.

**Table 3:** PCR primers used for amplification of genomic DNA and estimation of Fth1 copy numbers in *H. cumingii*.

Primers	(Primer sequence) (5′–3′)
gFER-F	GCTTTGCTGCGTCAGTGAAC
gFER-R	TTAGGGCTGGATCTTGTATCAATG
CNV-F	ATGATGTGGCCCTGAAAGGATTTG
CNV-R	CGGCCACCACGCTTGTTCTG
3′ UTR F	CTCAAGCGTGTTGGACCTGGACT
3′ UTR R	CAGCCCTATGACTTTTGAACAAGA

### Sequence analysis of full length cDNA

The alignment search tool BLASTn, from the National Center for Biotechnology Information was used to search homologous sequences in GenBank. The cDNA of ferritin was translated into its potential ORF by the ORF Finder algorithm (http://www.ncbi.nlm.nih.gov/gorf/). The putative amino acid sequences were analyzed for the presence of signal peptides using SignalP software (http://www.cbs.dtu.dk/services/SignalP/) [50]. Domain analyses were undertaken using several resources, including a Simple Modular Architecture Research Tool (http://smart.gembl-heidelberg.de/) [51], Pfam 20.0 (http://pfam.wustl.edu/) [52] and ScanProsite (http://www.expasy.org/tools/scanprosite/) [53]. Multiple sequence alignments were performed using ClustalW (http://www.ebi.ac.uk/Tools/clustalw2/index.html) and BOXSHADE (http://www.ch.embnet.org/software/BOX_form.html). SLS prediction was performed using the DINAMelt Web Server (Prediction of Melting Profiles for Nucleic Acids, http://mfold.rna.albany.edu/?q=DINAMelt/Quickfold) using default RNA3.0 parameters [54].

The phylogenetic tree was constructed based on the deduced full length amino acid sequences using the neighbor-joining algorithm in MEGA 4.0 [55], and the reliability of the analysis was assessed with 1000 bootstrap replicates.

### Copy number estimation

Two pairs of primers were designed according to the ferritin cDNA sequence using the EditSeq program in DNA Star 7 (DNASTAR, Inc., Madison, WI). One pair of primers was anchored in the 3′ UTR as a control (diploid copy numbers of the locus = 2) and the other pair was anchored in the conserved coding region of exon 2 (Table 3). Optimized primer pairs were selected based on their amplification efficiencies and specificities [56]. Amplification efficiencies were calculated based on the generation of standard curves using genomic DNA dilution series according to the equation: E = (10^−1/slope^−1)100 [57].

The amplification efficiencies were 97% and 98% for the 3′ UTR and CNV primer pairs respectively. A correction for different amplification efficiencies was introduced in the sample quantification process [58]. Melting curve analysis was used to check the specificities of PCR reactions.

Real-time PCR was performed with Bio-Rad IQ™5 Multicolor Real-Time PCR Detection System in a 25 µL reaction system containing the following components: 4 µL DNA sample, 12.5 µL 2×SYBR Green Supermix (Bio-Rad), 0.5 µL of each primer and 7.5 µL ddH_2_O. Briefly, following denaturation at 95°C for 10 min, real-time PCR was performed, with 40 cycles at a melting temperature of 95°C for 15 s, an annealing temperature of 60°C for 30 s and an extension temperature of 72°C for 30 s. Finally, a melting curve analysis was undertaken. All samples were amplified in triplicate from the same DNA preparation and the mean value was determined. The ferritin copy numbers were calculated using the comparative CT method according to the equation: diploid copy number = 2*2^(−(Ct^_Target gene_^−Ct^_Control gene_^))^. Cut-off values of 0.3 and 0.7 were used to define the copy numbers.

### Analysis of association between CNVs and growth

The statistical analysis was performed using SPSS 17.0 (SPSS, Inc., Chicago, IL). In the cultured population, three and four CNVs were the principal genotypes; other CNVs were less common (Fig. 5). Differences in growth (shell length, shell height and body weight) between individuals with three and four CNVs were assessed using the t-test. *P*-values were considered statistically significant if <0.05.
